# Transcriptomic and biochemical analyses revealed the dynamic regulatory mechanisms underlying leaf morphogenesis in *Camellia sinensis*

**DOI:** 10.3389/fpls.2026.1908225

**Published:** 2026-08-11

**Authors:** Xiang Liu, Linghong Zhou, Can Xu, Sisi Chen, Caiying Ou, Yu Fu, Ziyu Wang, Yong Li, Yong Luo

**Affiliations:** 1 School of Chemistry and Environmental Science, Xiangnan University, Chenzhou, Hunan, China; 2 Chenzhou Institute of Agricultural Sciences, Chenzhou, Hunan, China

**Keywords:** biochemical analysis, leaf development, regulatory mechanisms, tea plant, transcriptomic sequencing

## Abstract

Tea leaf development is a biological process marked by sequential morphogenesis that directly affects tea yield and quality. Despite their importance, the dynamic regulatory mechanisms governing these developmental stages remain poorly understood. In this study, we utilized tea buds, first leaves, second leaves, and third leaves from tea plants as experimental materials. By integrating transcriptomic sequencing with biochemical analysis, we systematically elucidated the transcriptional-metabolic co-regulatory network underlying leaf morphogenesis. The findings revealed significant differences in the leaf transcriptomes across developmental stages, with the most pronounced differentiation observed between buds and mature leaves. The comparison of bud vs. 3^rd^ leaf identified the highest number (6, 854) of differentially expressed genes, which were primarily concentrated in the KEGG metabolic pathways. Core metabolites, such as catechins, free amino acids, and alkaloids, were predominantly enriched in buds and young leaves, with most exhibiting a significant decline as the leaves matured. However, *O*-methylated catechins were significantly enriched in mature leaves. The expression of these core metabolites is regulated by differentially expressed genes in the phenylpropanoid and flavonoid pathways (*CHS*, *ANS*, *SCPL*, *OMTs*, etc.), theanine biosynthesis genes (*ALaDC*, *ALaAT*, *TS*, etc.), and purine alkaloid biosynthesis genes (*IMPDH*, *SAMS*, *TCS*, etc.). Correlation analysis indicated that most free amino acids (L-The, Glu, Gln, Asp, Ala, and GABA) exhibited significant positive correlations with alkaloids and catechins (C, GC, CG, EC, and ECG), whereas correlations with *O*-methylated catechin components were weak. Real-time quantitative PCR analysis revealed that 108 genes were significantly differentially expressed, with functions primarily involving cell wall synthesis and remodeling (*CER3*, *PRP4*, *EXPA1*, etc.), photosynthesis (*ndhL*), plant hormone signaling regulation (*SAUR23*, *CYP85A1*, *IAA14*, etc.), cell cycle (*CYCD1-1*, *CYCA2-4*), signal transduction (*MKK6*, *MLO6*), defense and secondary metabolism (*STR1*), and transcription factors (GRF1, MYB17, bHLH94, etc.). Furthermore, the promoter regions of functional genes related to leaf development showed significant enrichment of MYB and MYC transcription factors, as well as cis-regulatory elements for hormones (ABRE, ERE, TCA-element), light (GT1-motif, GATA-motif, TCT-motif), and stress signals (ARE, STRE, W box). This study identified the core genes and metabolic mechanisms underlying leaf morphogenesis in tea plants, providing a theoretical basis for high-quality cultivation of tea.

## 1 Introduction

Tea plants are economically important perennial crops with a global distribution and are renowned for their rich cultural heritage and distinctive flavor profiles (Jiang and Wen, 2025). Tea leaves are the primary raw material for tea production. Their development encompasses not only cell division, tissue differentiation, and maturation, but also the biosynthesis and accumulation of critical secondary metabolites that significantly influence tea yield and quality (Zhao et al., 2026; Xu et al., 2026). Accordingly, tea leaf morphogenesis directly determines both beverage quality and economic value. Previous studies have demonstrated that during tea leaf development, morphogenesis and functional stability arise from the biosynthesis and accumulation of specific metabolites, in conjunction with the coordinated regulation of related genes. Nevertheless, the dynamic regulatory mechanisms governing tea leaf morphogenesis remain incompletely understood (Wang et al., 2022b, 2025).

The developmental transition of tea leaves from morphological differentiation to physiological maturation constitutes a strictly programmed biological process under temporal three-stage hierarchical regulation, which can be divided into initiation, primary morphogenesis, and secondary morphogenesis. This process is co-modulated by a complex interplay of multiple genes, signaling pathways, metabolic networks, environmental factors, and cultivation practices (Xu et al., 2026). This progression, consistent with that observed in other plants, strictly follows the hierarchical regulation of leaf primordium initiation, morphogenetic development, and maturation stages, encompassing leaf cell division, elongation, expansion, and differentiation of secondary structures, such as veins and trichomes (Dengler and Tsukaya, 2001; Bar and Ori, 2014; Du et al., 2018). At the molecular level, gene regulation, signaling cascades, and metabolic pathways operate in an integrative manner. For example, MYB and bHLH transcription factors form the MBW complex (MYB-bHLH-WD40), a central regulatory module that drives the biosynthesis of metabolites, including anthocyanins, catechins, and alkaloids (Hu et al., 2024; Zhang et al., 2026). Additionally, bHLH factors either function independently or interact with WRKY and NAC transcription factors to co-regulate defense responses and maintain leaf physiological homeostasis (Liu et al., 2024; Han et al., 2025; Xie et al., 2026), whereas TCP transcription factors modulate *MYB* expression, linking morphogenesis with secondary metabolism (Yu et al., 2021). Hormonal signaling further integrates these networks, as auxin regulates MYB factors to direct polarized leaf growth (Li et al., 2022), and gibberellin influences TCP factors to modulate cell elongation and expansion (Wu et al., 2017), thereby ensuring orderly leaf development across growth stages and environments. Moreover, environmental and agronomic factors, including light intensity, temperature, water availability, altitude, pruning, and fertilization, significantly influence morphological and physiological maturation by modulating leaf architecture, function, and metabolite accumulation (Zhang et al., 2025a; Qiu et al., 2024; Li et al., 2024b).

In recent years, the relationship between gene expression and metabolite accumulation has been preliminarily elucidated at both the molecular and metabolic levels, with significant advancements achieved in tea plant research. For instance, integrated single-cell transcriptomic and metabolomic analyses have delineated the developmental trajectory of tea plants from bud to leaf, elucidating the temporal accumulation patterns of characteristic metabolites, such as catechins and flavonoids, alongside their principal regulatory pathways. This approach offers a novel single-cell perspective for investigating the molecular mechanisms underlying leaf morphogenesis and functional maturation in tea plants (Zhao et al., 2026). Furthermore, multi-omics studies have uncovered the molecular basis of quality variations in tea leaves across different developmental stages (Wang et al., 2023; Chen et al., 2024). For instance, Wang et al. (2023) employed the tea cultivar ‘Sichuan Colonial’ as experimental material to profile the metabolic dynamics of six catechin monomers, two alkaloids, and theanine across tea leaves with distinct developmental phases, and identified core regulatory genes governing these metabolites via protein–protein interaction network analysis. In a follow-up study, Chen et al. (2024) selected the tea cultivar ‘Huangjinye’ to characterize metabolic variations in eight catechin components, one alkaloid, and three free amino acids during leaf ontogeny and preliminarily excavated candidate transcription factors responsible for modulating these metabolic substances. Our previously research deciphered the tissue-specific accumulation characteristics of *O*-methylated EGCG throughout leaf developmental gradients and systematically dissected the MYB-mediated regulatory cascade underlying the biosynthesis of *O*-methylated catechins at different leaf developmental stages (Luo et al., 2025). However, systematic and comprehensive investigations into the regulatory mechanisms governing the entire developmental progression of tea leaves, from morphogenesis to functional maturation, remain insufficient. The mechanisms orchestrating the coordinated interplay between transcriptional regulation and biochemical metabolism during leaf development have not yet been fully elucidated. Moreover, key regulatory genes, biochemical metabolic pathways, and their interaction networks involved in this developmental process lack thorough systematic analyses.

This study used tea plant leaves at various developmental stages as experimental material to systematically characterize and analyze the metabolic profiles and gene expression dynamics and correlations of 11 catechin derivatives, 4 alkaloids, and 37 amino acid components throughout leaf development. By comprehensively elucidating the synergistic regulatory interactions between gene expression and biochemical metabolism, and systematically analyzing the changes of major functional genes during leaf development, this study aimed to reveal the dynamic regulatory network governing the transition of tea leaves from morphogenesis to functional maturation. These findings will advance our understanding of the molecular regulatory mechanisms underlying this developmental process and have significant theoretical and practical implications for optimizing high-quality tea cultivation practices.

## 2 Materials and methods

### 2.1 Plant materials

The tea plant variety used in this study was Lingtou Dancong (LTDC), which was cultivated in Yingde County, Qingyuan City, Guangdong Province (24^°^10′N, 113^°^22′E). This variety is characterized by high levels of catechins and *O*-methylated catechins. Fresh tissues at different developmental stages, including tea buds (B), first leaf (L1), second leaf (L2), and third leaf (L3), were selected for the study, and samples were collected in April 2024. Fresh tissues were immediately frozen in liquid nitrogen after collection and stored at -80 °C. Each biochemical experiment was performed in triplicate.

### 2.2 Determination of catechins and alkaloids

The detection conditions for catechins and alkaloids in tea were optimized according to GB/T 8313–2018 and Wang et al. (2022a). Accurately weigh 0.1 g of freshly ground, homogenized tea powder into a 2 mL centrifuge tube, add 1 mL of 70% methanol solution, and perform ultrasonic extraction in an ice-water bath for 20 min. After centrifugation at 12, 000 rpm/min for 5 min, the supernatant was filtered through a 0.22 μm membrane, diluted tenfold with 70% methanol, and subjected to analysis. catechins and alkaloids were analyzed using a high-performance liquid chromatography (HPLC) system (Alliance, Waters, Milford, MA, USA) equipped with a ZORBAX Eclipse C18 column (4.6 mm × 150 mm, 5 μm). The injection volume was 10 μL, and the column temperature was maintained at 40 °C. Mobile phase A consisted of distilled water containing 7% acetonitrile and 2% glacial acetic acid, and mobile phase B consisted of acetonitrile and 2% glacial acetic acid. The solvent linear gradient was: 0–4.8 min, 97% A; 4.81 min, 100% A; 4.81–19 min, 100% A; 19.01–21 min, 90% A; 21.01–31 min, 82% A; 31–31.01 min, 80% A; 31.01–33 min, 75% A; 33.00–33.01 min, 100% B; 33.01–63 min, 100% B; 63–63.1 min, 97% A; 63.1–93 min, 97% A, with a flow rate of 1 mL/min. The UV absorption wavelength was set to 278 nm. Ultraviolet spectral identification was performed using a diode array detector, and the compounds were qualitatively and quantitatively analyzed based on the standards.

### 2.3 Detection of free amino acids

The method for the extraction and determination of free amino acids in fresh tea leaf samples was adapted from a published article (Zeng et al., 2017) and the national standard GB/T 30987-2020, with some modifications: Weigh 100 mg of ground fresh tea leaf sample into a 2 mL centrifuge tube, add 0.5 mL of pre-chilled methanol, and vortex for 2 min; sonicate in an ice bath for 15 min; add 0.5 mL of chloroform and 0.2 mL of pre-chilled ultrapure water, vortex for 1 min to facilitate phase separation; centrifuge at 5000 g for 10 min, and collect 500 μL of the supernatant into a 2 mL centrifuge tube; concentrate the collected supernatant by vacuum drying at 45 °C using a rotary evaporator; add 1 mL of 3% sulfonated salicylic acid (prepared with 0.1 M hydrochloric acid) to resuspend the solid obtained from centrifugation and concentration, and let stand for 1 h; centrifuge at 5000 g for 10 min, then filter the supernatant through a 0.22 μm nylon membrane to obtain the sample solution for analysis. Theanine was diluted with Solution A, and free amino acids were detected using a high-performance sodium cation exchange column (4.0 mm × 150 mm, Pickering Laboratories, Inc., USA). The Sykam S433D Physiological Li C4 system was used with mobile phases containing lithium citrate (pH 2.9, 4.2, and 8.0), with UV-Vis detection set at 570 and 440 nm. The mobile phase flow rate was 7.5 μL/s, and the indanone (derivatization reagent) flow rate was 4.167 μL/s. The column temperature was 38 °C, and the reaction unit (post-column) temperature was 130 °C. The autosampler temperature was maintained at 5 °C, and the injection volume was 50 µL. A 3% solution of sulfosalicylic acid was prepared using 0.1 M HCl solution.

### 2.4 Transcriptome sequencing and analysis

Transcriptome sequencing was conducted by Guangzhou GidiO Biotechnology Co., Ltd. The specific procedure was as follows: Total RNA was extracted from leaf samples of various LTDC tissues; after removing impurities such as rRNA, mRNA was enriched; the enriched mRNA was reverse-transcribed into double-stranded cDNA; the cDNA was repaired at both ends, adapters were added, and PCR amplification was performed to construct the library for sequencing. After the library passed quality control, gene annotation was performed using the LTDC genome (Chen et al., 2023) for gene annotation. The fragments per kilobase of the exon model per million mapped fragments for each gene was calculated based on gene length, and the number of reads mapped to each gene was determined. DESeq2 R software (version 1.16.1) (Love et al., 2014) was used to perform differential expression analysis between the two groups. Genes with an adjusted p-value <0.05, identified by DESeq2, were designated as differentially expressed genes (DEGs). Venn diagrams were generated using the tool available at the following URL: https://www.biovenn.nl/index.php. For principal component analysis (PCA), a three-component model was selected for the distance measurement. The cluster Profiler R package was used to identify metabolic pathways associated with DEGs in the Kyoto Encyclopedia of Genes and Genomes (KEGG) database. Fragments per kilobase of transcript per million mapped reads was calculated to estimate genes expression levels.

### 2.5 Quantitative real-time reverse transcription PCR analysis

qRT-PCR was performed as previously described (Luo et al., 2025) using the LightCycler480 real-time PCR system (Roche, Basel, Switzerland) and Bio-Rad iTaq Universal SYBR Green Supermix kit (catalog number: 1725124) for real-time fluorescence quantitative PCR detection. The PCR cycling conditions were as follows: 95 °C for 3 min for 1 cycle; then 95 °C for 10 s and 60 °C for 30 s for 40 cycles; after the reaction, samples were heated from 60 °C to 95 °C at a rate of 0.2 s, and fluorescence was continuously recorded for melting curve analysis. Relative gene expression levels were analyzed using the 2^-ΔΔCt^ method. The primers used in this study are listed in **Supplementary Table 1**.

### 2.6 Cis-element analysis of functional genes

Cis-element analysis of gene promoters was performed using Plant Care (http://bioinformatics.psb.ugent.be/webtools/plantcare/html/). After statistical analysis of the number of different cis-regulatory elements, TBtools was used for visualization to demonstrate the number of different cis-regulatory elements present in various functional genes.

### 2.7 Statistical analysis

Correlations between substances were calculated, and their relationships were visualized using a correlation plot. Association analysis between substances was performed using Metware Cloud, a free online platform for data analysis (https://cloud.metware.cn). All data were derived from three independent biological replicates of each experiment. Data are presented as mean ± standard deviation (SD). Statistical analyses were conducted using the SPSS software (version 22.0; SPSS, Chicago, Illinois, USA). Significant differences between the treatment and control groups were calculated using Student’s *t*-test.

## 3 Results and discussion

### 3.1 Leaf development phenotypes and principal component analysis of RNA sequencing

**Figure 1:**
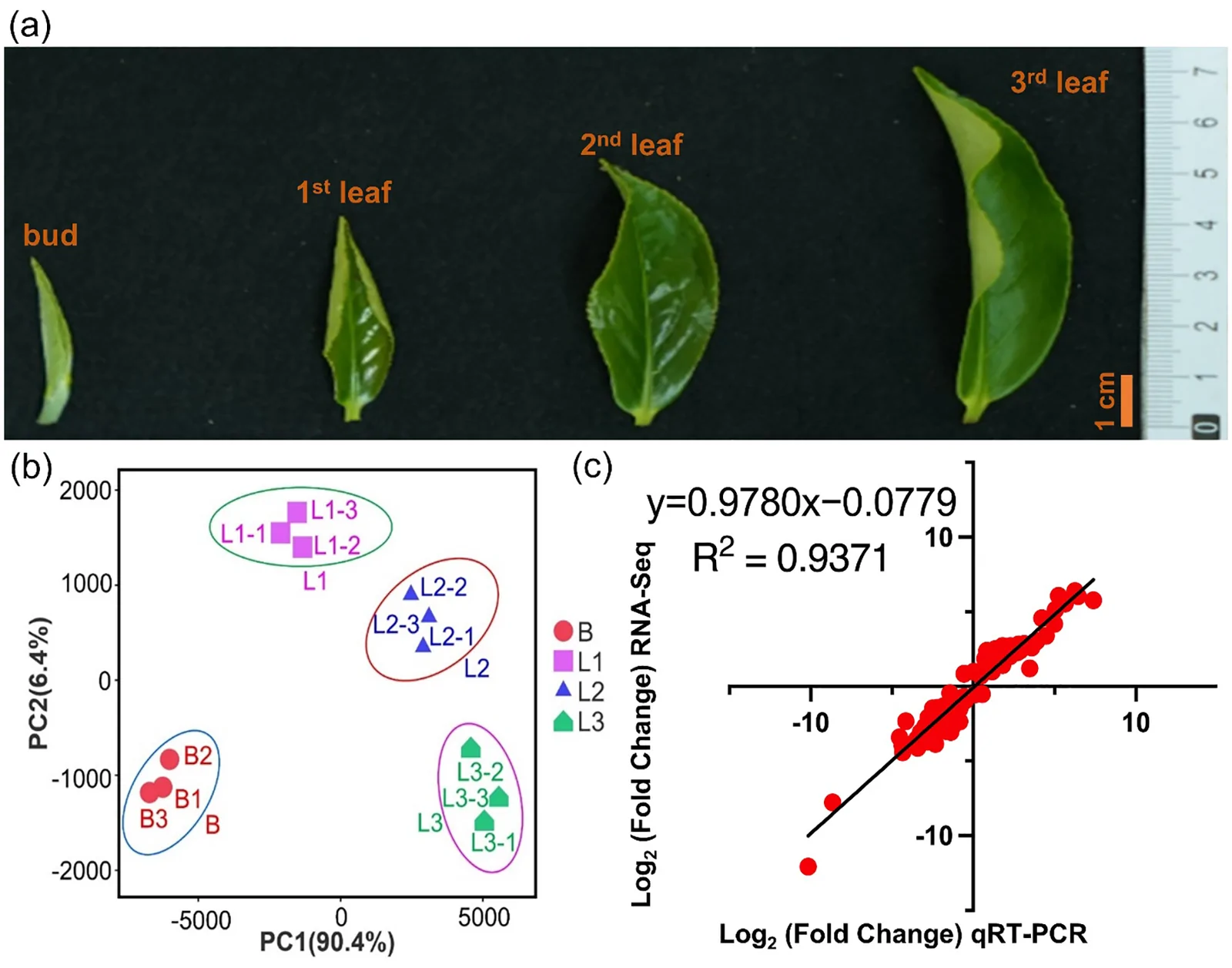
Phenotypic traits and RNA-seq-based PCA of tea leaves at four developmental stages. **(a)** Morphological phenotypes of leaves at four developmental stages. Scale bars = 1 cm. **(b)** PCA plot of samples B, L1, L2, and L3. Each colored ellipse encloses a sample group containing three biological replicates, and each dot indicates an individual sample. Sample clustering was performed using the transcriptome profiles. **(c)** Correlation analysis between qRT-PCR quantification and transcriptome sequencing results of the 30 screened candidate genes. PCA, principal component analysis; PC, principal component; B, tea buds; L1, 1st leaf; L2, 2nd leaf; L3, 3rd leaf.

Tea-leaf morphogenesis exhibits multidimensional characteristics. Morphologically, the leaves undergo multiple stages, including bud, curling, unfolding, and maturation (**Figure 1A**). B, L1, L2, and L3 shown in the figure illustrate the process of tea leaf morphogenesis. Additionally, tea leaves at different developmental stages exhibit significant differences at the metabolic and molecular levels. To further evaluate the dynamic regulatory mechanisms underlying the morphological development of tea leaves, transcriptomic sequencing was performed on tea leaves at different developmental stages. As shown in **Figure 1B**, each sample group (B, L1, L2, and L3) had three replicate RNA-seq datasets. PCA revealed that each variety group (B, L1, L2, and L3) was enclosed by differently colored ellipses in the PCA output. PC1 and PC2 explained 90.4% and 6.4% of the total variance, respectively, with a cumulative contribution of 96.8%, effectively reflecting the transcriptional differences among the samples. Furthermore, in the PCA score plot, different tissue samples exhibited distinct intra-group clustering and inter-group separation: Group B was concentrated on the negative half-axis of PC1, clearly distinguishing them from other leaf tissues; Groups L1, L2, and L3 were distributed sequentially from left to right along the PC1 axis, and the three biological replicates within each group were tightly clustered, indicating good reproducibility. These results indicate significant differences in transcriptional levels among tea plant leaves at different developmental stages. The differences in transcriptional profiles between B and mature leaves (L3) were the most pronounced, whereas certain differences also existed among leaves at different developmental stages (L1, L2, and L3). This suggests dynamic regulation during tea plant leaf morphogenesis, providing a fundamental basis for further elucidating the dynamic regulatory mechanisms underlying tea leaf morphogenesis. To verify the reliability of the RNA-seq data, 30 samples were randomly selected from the transcriptome dataset for qRT-PCR validation of DEGs. The qRT-PCR results exhibited a strong positive correlation with RNA-seq data. A high coefficient of determination (R^2^ = 0.9371) was obtained between the two datasets, thereby confirming the robustness and credibility of the RNA-seq transcriptomic data (**Figure 1C**).

### 3.2 DEG analysis

**Figure 2:**
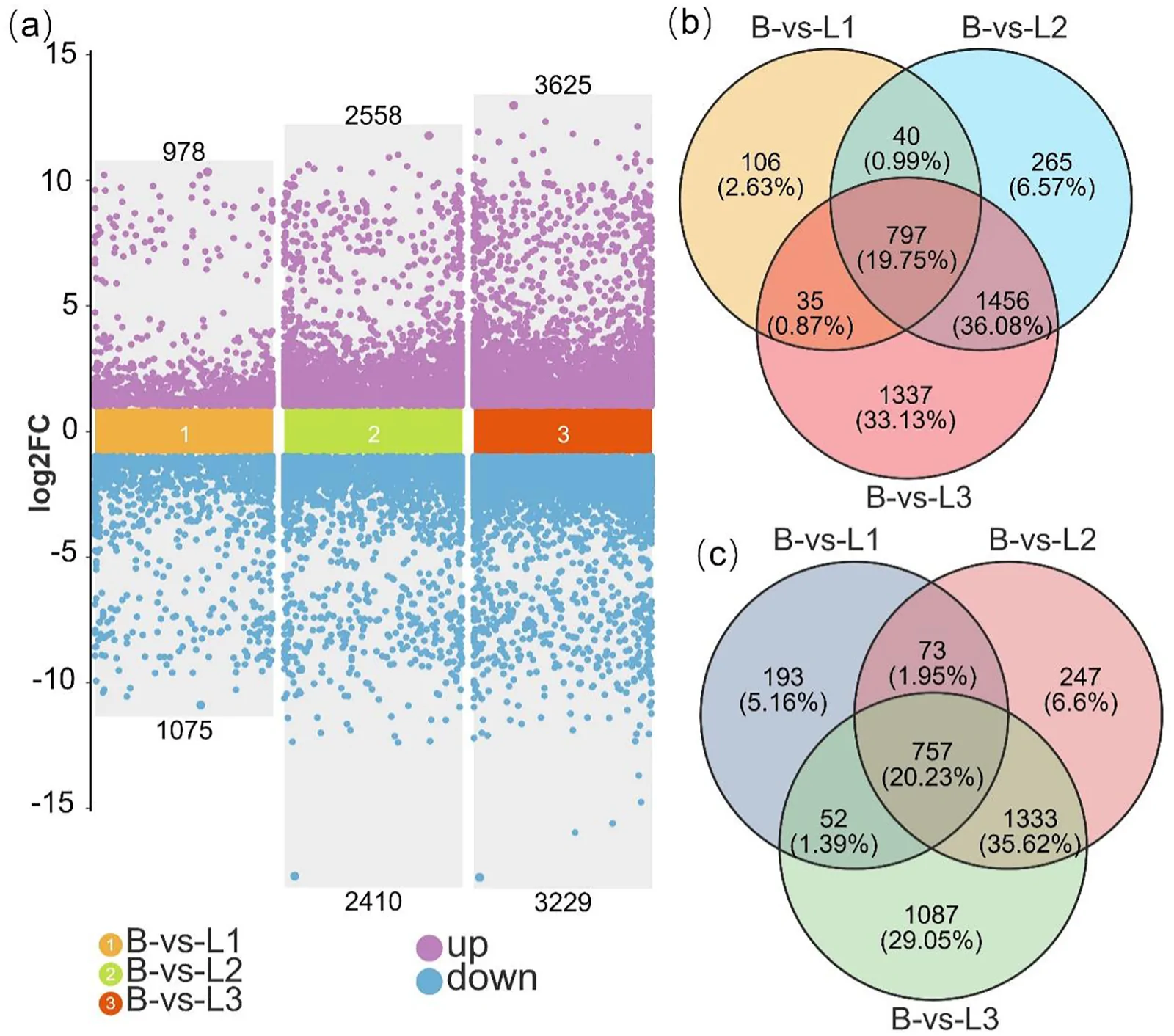
Statistics of DEGs identified from the three transcriptome comparison groups (B vs. L1, B vs. L2, and B vs. L3). **(a)** Number of upregulated and downregulated DEGs in each comparison. **(b)** Venn diagram showing the overlap of upregulated DEGs. **(c)** Venn diagram showing the overlap of downregulated DEGs. DEGs, differentially expressed genes.

DEGs at different developmental stages of tea leaves were identified using thresholds of FDR < 0.05 and |log_2_fold change| > 2. In pairwise comparisons between B vs L1, B vs L2, and B vs L3, the results showed that compared to group B, the total number of DEGs increased significantly with leaf maturity. The numbers of upregulated genes were 978, 2, 558, and 3, 625, respectively, and there were 1, 075, 2, 410, and 3, 229 downregulated genes, respectively (**Figure 2A**). The highest number of DEGs was observed between B vs L3 (6, 854), indicating that the transcriptional differences between B and L3 gradually widened as leaf development progressed. A Venn diagram was constructed to illustrate the distribution of sample-specific and shared DEGs among the different samples. As shown in the Venn diagram, the three pairwise comparisons (B vs. L1, B vs. L2, and B vs. L3) shared 797 differentially upregulated genes (**Figure 2B**) and 757 differentially downregulated genes (**Figure 2C**), accounting for 19.75% and 20.23% of the total, respectively. In addition, each group contained unique DEGs, with the B-vs-L3 comparison exhibiting the highest number of unique DEGs (1, 337 upregulated and 1, 087 downregulated), reflecting the specific characteristics of gene expression remodeling during the late stages of leaf maturation (**Figures 2B, C**). These results indicate that gene expression undergoes dynamic changes during tea leaf development, featuring both core DEGs present throughout the entire developmental process and transcriptional regulation specific to the maturation stage, thereby providing data support for the subsequent identification of key regulatory genes involved in tea leaf morphogenesis.

**Figure 3:**
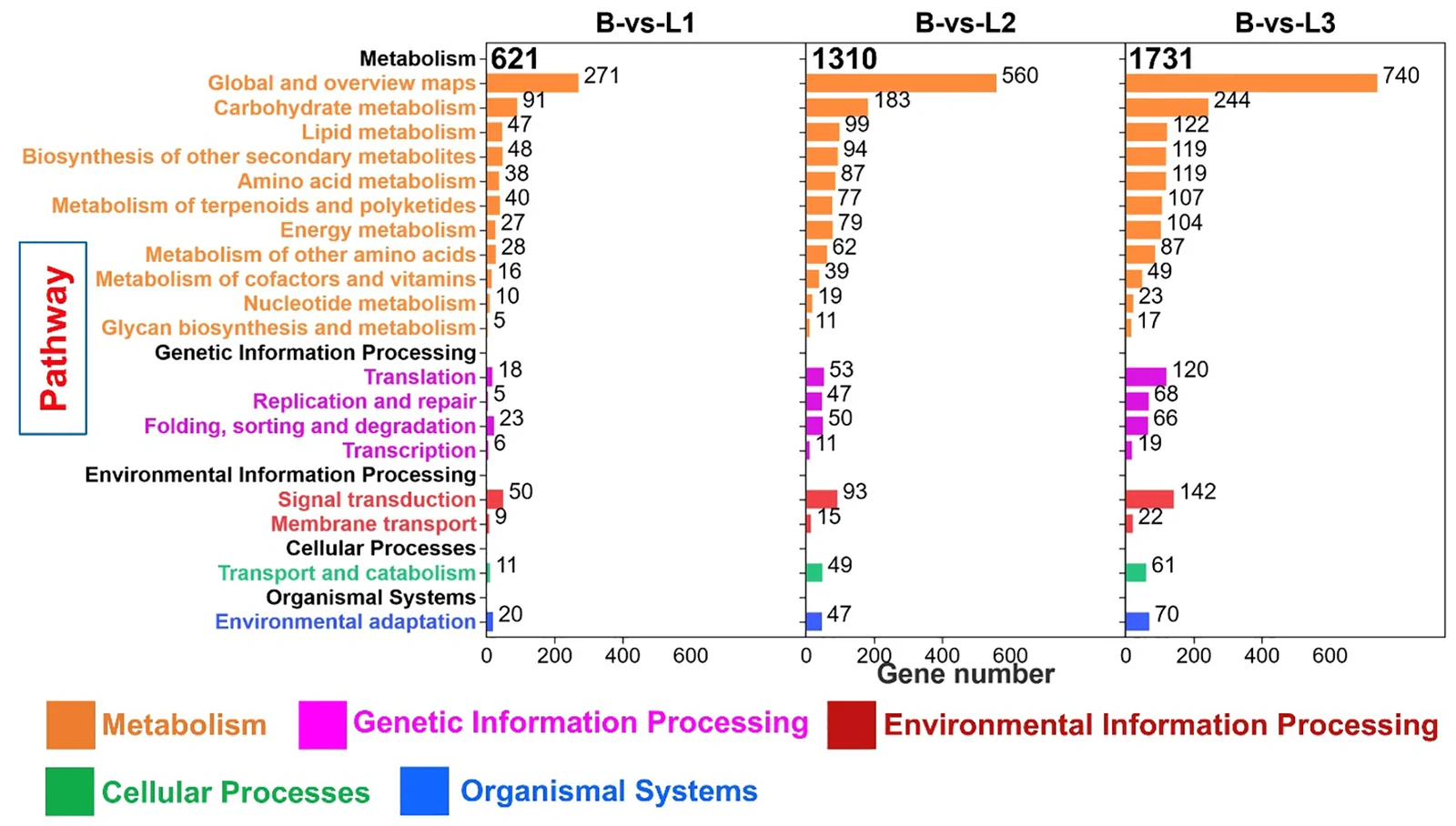
KEGG pathway enrichment analysis of DEGs in tea leaves at four developmental stages.

To analyze the functional distribution of DEGs between B and leaves at different developmental stages (L1, L2, and L3), pathway annotation analysis was performed using the KEGG database. The results (**Figure 3**) showed that the DEGs across the three comparisons were primarily enriched in the metabolism pathway, with the number of genes annotated to this pathway increasing significantly with leaf maturity, with 621 genes annotated to B vs L1, 1310 genes annotated to B vs L2, and 1731 genes annotated to B vs L3, respectively. The most significant enrichment was observed in genes related to global metabolism, carbohydrate metabolism, lipid metabolism and secondary metabolite biosynthesis. In addition to metabolic pathways, differential gene enrichment was also found in Genetic Information Processing and Environmental Information Processing pathways, with the number of enriched genes similarly increasing with leaf maturity. These findings suggest active gene expression regulation and signal transduction during the development of leaves. Overall, gene expression changes in metabolism-related pathways were most pronounced during the transition from B to L3. The dynamic regulation of genes involved in secondary metabolite biosynthesis likely drives metabolic differences among leaves at different maturity stages, providing crucial information for identifying key functional genes.

### 3.3 Changes in catechins and related genes during leaf morphogenesis

**Figure 4:**
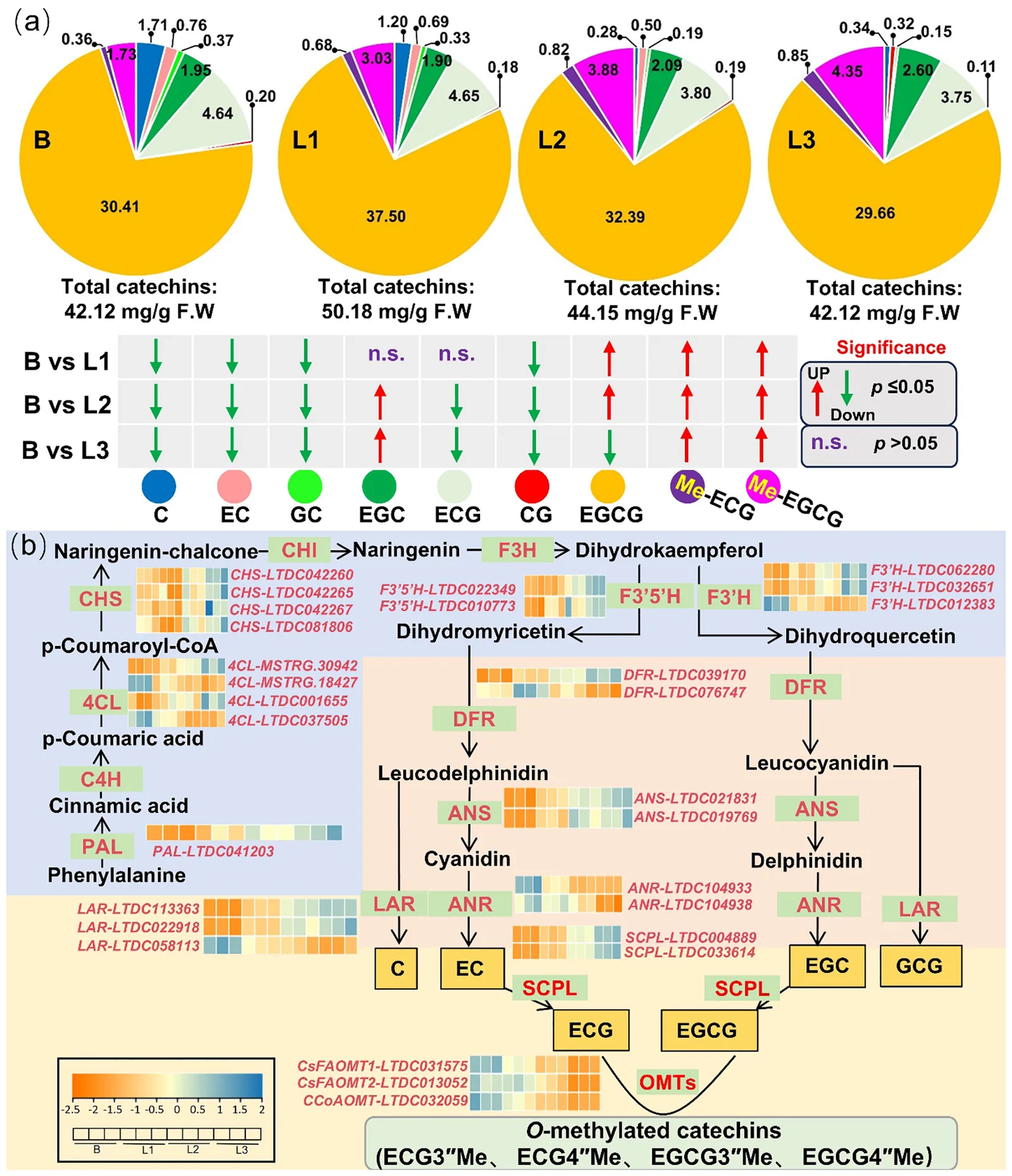
Accumulation of catechin components and expression profiles of catechin biosynthesis-related genes in tea leaves at four developmental stages. **(a)** Total catechin content and individual catechin monomers. Data are presented as mean ± SD (n = 3). Significant differences between treatment and control groups were calculated by Student’s t-test. “
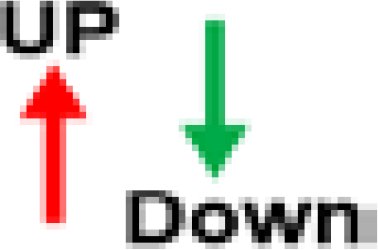
” indicate significantly upregulated or downregulated expression relative to the control group (*p ≤*0.05), respectively. While “n.s.” represents no significant differences (*p >*0.05). **(b)** Schematic representation of the catechin metabolic pathway and expression patterns of associated genes during leaf development. Fragments Per Kilobase of transcript per million sequenced reads (FPKM) values were computed to evaluate the relative expression abundance of individual genes. C, (+)-catechin; EC, (−)-epicatechin; GC, (+)-gallocatechin; EGC, (−)-epigallocatechin; ECG, (−)-epicatechin gallate; CG, catechin gallate; EGCG, (−)-epigallocatechin gallate; Me-ECG, total *O*-methylated ECG (ECG3″Me and ECG4″Me); Me-EGCG, total *O*-methylated EGCG (EGCG3″Me and EGCG4″Me). Enzymes: PAL, Phenylalanine ammonia-lyase; C4H, Cinnamate 4-hydroxylase; 4CL, 4-Coumarate-CoA ligase; CHS, Chalcone synthase; CHI, Chalcone isomerase; F3H, Flavanone 3-hydroxylase; F3’H, Flavonoid 3’-hydroxylase; F3’5’H, Flavonoid 3’, 5’-hydroxylase; DFR, Dihydroflavonol 4-reductase; ANS, Anthocyanidin synthase; LAR, Leucoanthocyanidin reductase; ANR, Anthocyanidin reductase; SCPL, Serine carboxypeptidase-like acyltransferase; OMTs, *O*-methyltransferases. F.W, Fresh Weight.

A systematic analysis was conducted on the catechin content in B and leaves at different maturity levels (L1, L2, and L3). Quantitative results for catechin components (**Figure 4A**) showed significant differences in total catechin content among leaves at various developmental stages, exhibiting a trend of first increasing and then decreasing. The content was lowest during the B and L3 stages (42.12 mg/g F.W), whereas the highest total catechin content was observed at the L1 stage (50.18 mg/g F.W). From the L2 to L3 stages, the total catechin content decreased, indicating that catechin biosynthesis and accumulation exhibit distinct developmental stage specificity. Analysis of catechin composition revealed that EGCG was the most abundant catechin component in all tissues, followed by ECG. Statistical analysis showed that compared with B, most catechins (C, EC, GC, ECG, and CG) exhibited a significant downward trend in the L1, L2, and L3 stages, whereas *O*-methylated catechins (Me-ECG and Me-EGCG) were significantly upregulated. This suggests that catechin biosynthesis is more active in buds and young leaves, whereas the activity of methylated catechin synthesis increases as leaf maturity.

Further gene expression analysis of the catechin biosynthetic pathway (**Figure 4B**) showed that key genes in the phenylpropanoid and flavonoid biosynthesis pathways (such as *PAL*, *C4H*, *4CL*, *CHS*, *CHI*, *F3H*, *F3’H*, *DFR*, *LAR*, *ANS*, *ANR*, *SCPL*, and *OMTs*) exhibited significant differences in expression during developmental stages of leaves. In bud tissue, *LAR* (*LTDC113363* and *LTDC022918*), *ANS*, *DFR* (*LTDC039170*), and *SCPL* (*LTDC004899* and *LTDC033614*)—key genes catalyzing catechin modification and biosynthesis—exhibited the highest expression levels, which decreased with increasing leaf maturity, consistent with the high accumulation of most catechins (C, EC, GC, CG, and EGCG) in buds. In contrast, the expression levels of *DFR* (*LTDC076747*), *LAR* (*LTDC058113*), *ANR*, and *OMTs* were significantly upregulated in L3, consistent with the high accumulation of certain catechins (such as EGC) and *O*-methylated catechins (Me-ECG and Me-EGCG) in L3. Meanwhile, the differential expression of upstream phenylpropanoid genes (such as *PAL*, *C4H*, *4CL*, *CHS*, *F3’5’H*, and *F3’H*) provided ample precursor materials for subsequent flavonoid biosynthesis. This suggests that the dynamic changes in catechin composition during tea leaf development result from the coordinated regulation of the differential expression of key genes in the synthetic pathways.

### 3.4 Changes in free amino acids and related genes during leaf morphogenesis

**Figure 5:**
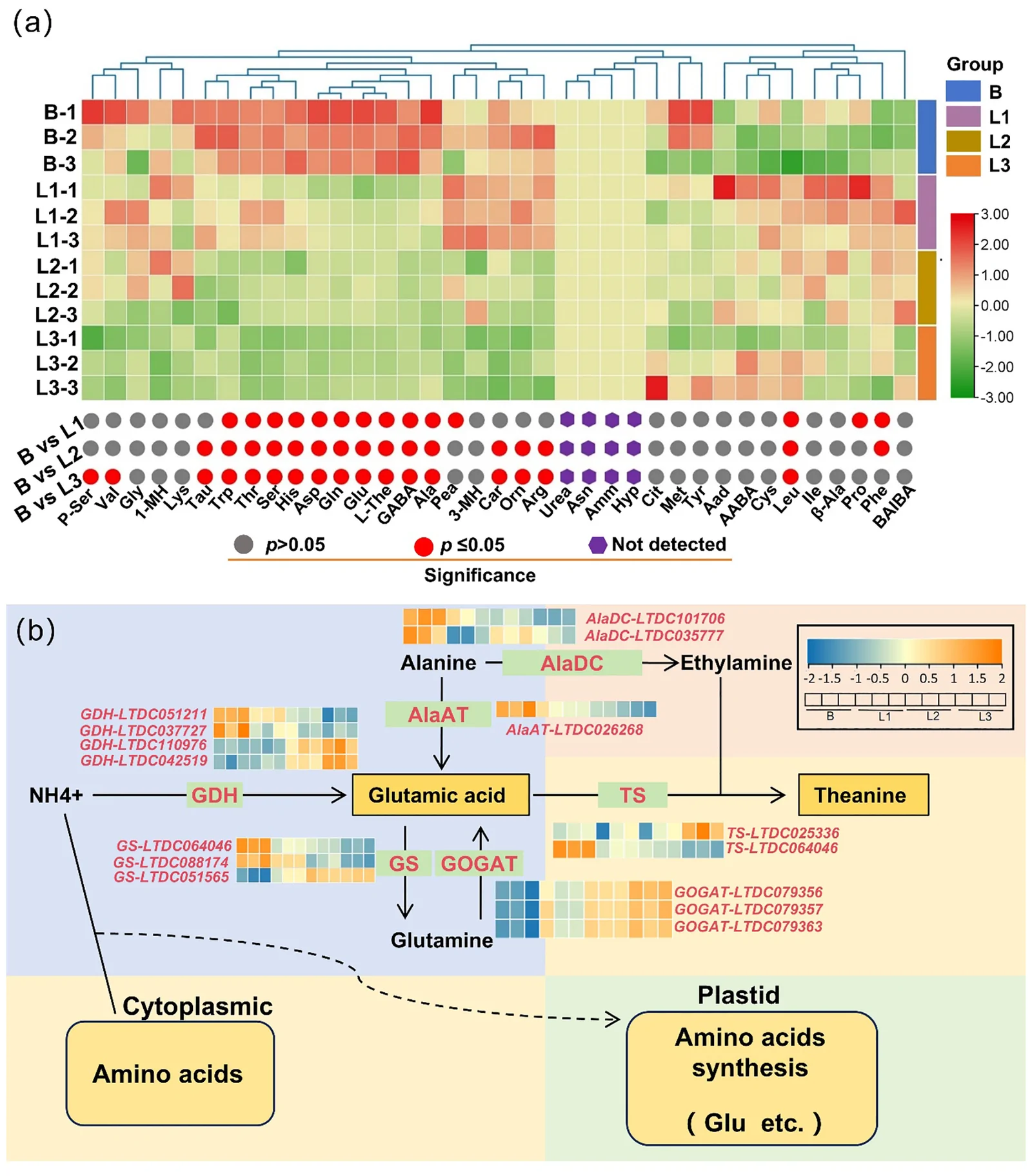
Dynamic changes in free amino acid content and expression of genes involved in amino acid metabolism in tea leaves at four developmental stages. **(a)** Contents of free amino acids detected in leaf samples. Data are presented as mean ± SD (n = 3). Significant differences between treatment and control groups were calculated by Student’s t-test. “
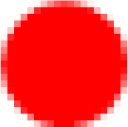
” indicate significantly different relative to the control group (*p ≤*0.05), while “
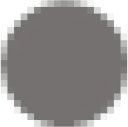
” represents no significant differences (*p >*0.05). “
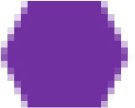
” represents not detected. **(b)** Schematic diagram of free amino acid metabolic pathways and expression patterns of related functional genes during leaf development. Fragments Per Kilobase of transcript per million sequenced reads (FPKM) values were computed to evaluate the relative expression abundance of individual genes. P-Ser, Phosphoserine; Val, Valine; Gly, Glycine; 1-MH, 1-methylhistidine; Lys, Lysine; Tau, Taurine; Trp, Tryptophan; Thr, Threonine; Ser, Serine; His, Histidine; Asp, Aspartic acid; Gln, Glutamine; Glu, Glutamic acid; L-The, Theanine; GABA, γ-aminobutyric acid; Ala, Alanine; Pea, Phosphoethanolamine; 3-MH, 3-methylhistidine; Car, Carnosine; Orn, Ornithine; Arg, Arginine; Asn, Asparagine; Amm, Ammonium chloride; Hyp, Hydroxyproline; Cit, Citrulline; Met, Methionine; Tyr, Tyrosine; Aad, α-aminohexanedioic acid; AABA, α-aminobutyric acid; Cys, Cystine; Leu, Leucine; Ile, Isoleucine; β-Ala, β-alanine; Pro, Proline; Phe, Phenylalanine; BAIBA, β-Aminoisobutyric acid; GDH, Glutamate dehydrogenase; AlaAT, Alanine aminotransferase; AlaDC, Alanine decarboxylase; TS, Theanine synthetase; GS, Glutamine synthetase; GOGAT, Glutamine 2-oxoglutarate aminotransferase. F.W, Fresh Weight.

We conducted a systematic analysis of the content of 37 free amino acid components and the L-theanine (L-The) biosynthetic pathway in buds and leaves at different stages of maturity (L1/L2/L3). The amino acid expression heatmap (**Figure 5A**) shows significant differences in the amino acid profiles of leaves at different developmental stages. In group B, the levels of most free amino acids, such as L-The, its precursors glutamic acid (Glu), glutamine (Gln), and alanine (Ala), tryptophan (Trp), threonine (Thr), serine (Ser), histidine (His), aspartic acid (Asp), and gamma-aminobutyric acid (GABA), were significantly higher than those in leaves (L1–L3) and showed a clear downward trend in leaf samples. Some amino acids, including Urea, asparagine (Asn), ammonia (Amm), and hydroxyproline (Hyp), were undetectable in both buds and leaves. Additionally, leucine (Leu) accumulated in the leaves, suggesting that amino acid11 accumulation exhibits distinct tissue-specificity.

Further gene expression analysis of theanine biosynthetic pathway (**Figure 5B**) revealed significant differences in the expression of key genes across tissues. *GDH-LTDC051211*, *GDH-LTDC037727*, and *AlaAT-LTDC026268* had the highest expression levels in bud tissue and showed a decreasing trend with leaf development, supplying abundant glutamate precursors for the accumulation of L-The. The L-The synthase gene *TS-LTDC064046* and ethylamine synthase genes *AlaDC-LTDC101706* and *AlaDC-LTDC035777* also showed high expression in buds, consistent with elevated theanine levels in buds. Additionally, genes such as *GOGAT*, *GS* (*LTDC051565*), and *GDH* (*LTDC110976*, *LTDC042519*) were upregulated during leaf development, indicating that leaves may serve as a nitrogen metabolism source, exhibit intense photorespiration and high demands for ammonia recycling and nitrogen transport. This necessitates the involvement of genes such as *GOGAT*, *GS*, and *GDH*, which is consistent with previous findings (Liu et al., 2021). These results elucidate the developmental regulation of amino acid metabolism in tea leaves and provide a theoretical foundation for enhancing tea freshness and flavor via targeted gene regulation.

### 3.5 Changes in alkaloids and related genes during leaf morphogenesis

**Figure 6:**
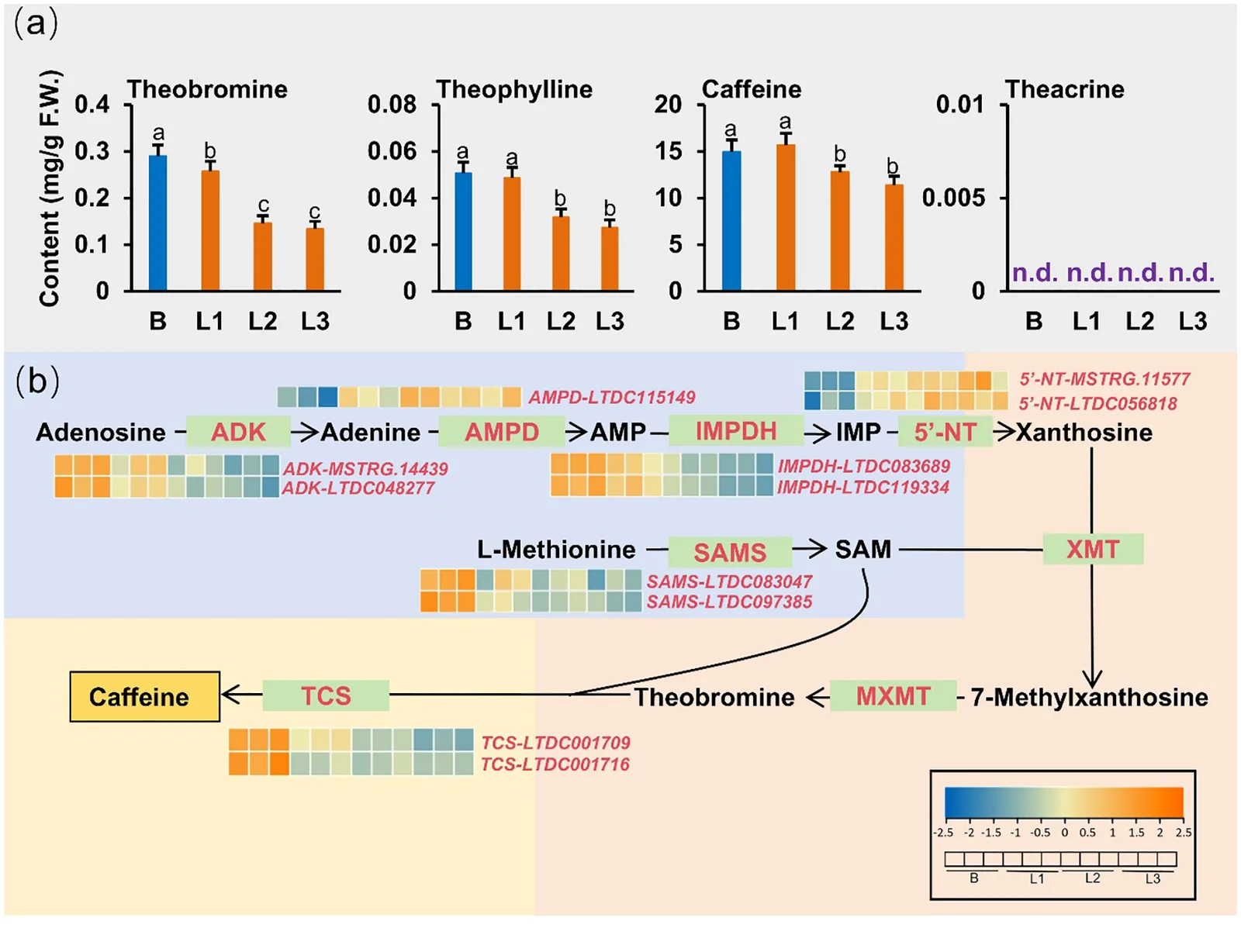
Alkaloid accumulation and expression of alkaloid biosynthesis genes in tea leaves at four developmental stages. **(a)** Alkaloid content in the leaf samples. Data are presented as mean ± SD (n = 3). Means distinguished by different letters are significantly different from each other (*p* ≤ 0.05). “n.d.” represents not detected. **(b)** Schematic representation of the alkaloid metabolic pathway and expression patterns of related genes. Fragments Per Kilobase of transcript per million sequenced reads (FPKM) values were computed to evaluate the relative expression abundance of individual genes. ADK, Adenosine kinase; AMPD, Adenosine monophosphate deaminase; IMPDH, Inosine monophosphate dehydrogenase; 5’-NT, 5’-Nucleotidase; XMT, Xanthosine methyltransferase; SAMS, S-adenosylmethionine synthetase; TCS, Tea caffeine synthase; MXMT, 7-Methylxanthine methyltransferase. F.W, Fresh Weight.

We analyzed the purine alkaloid content in buds and leaves at different stages of maturity (L1, L2, and L3) and conducted a systematic analysis of the caffeine biosynthesis pathway. The results of content determination (**Figure 6A**) showed significant differences in the levels of theobromine, theophylline, and caffeine in leaves at different developmental stages, with all three exhibiting a clear downward trend as leaf maturity increased. The levels of all three alkaloids were highest in bud tissue, with caffeine reaching approximately 15 mg/g F.W. Levels rose slightly in the L1 stage but without significant difference, while they decreased significantly in the L2 and L3 stages. The trends for theobromine and theophylline were consistent with those of caffeine, with levels in the L2 and L3 stages significantly lower than those in the B and L1 stages. However, theacrine was not detected in any of the samples (n.d.). Statistical analysis revealed that, compared to stages L2 and L3, stage B exhibited significant differences in the levels of theobromine, theophylline, and caffeine (p < 0.05), indicating that the biosynthesis and accumulation of purine alkaloids exhibit distinct developmental stage specificity, with buds and young leaves serving as the primary sites for caffeine biosynthesis and storage.

Further gene expression analysis of the caffeine biosynthetic pathway (**Figure 6B**) revealed that key genes involved in this pathway (*ADK*, *AMPD*, *IMPDH*, *SAMS*, *XMT*, *MXMT*, and *TCS*) exhibited significant differences in expression across various tissues. Among these, the upstream genes related to adenine biosynthesis (e.g., *ADK*), the *SAMS* gene that provides a methyl donor, and the genes encoding key enzymes catalyzing caffeine biosynthesis (*TCS* and *IMPDH*) all showed high expression levels in buds, consistent with the elevated accumulation of caffeine and theobromine in the buds. During leaf development, the expression levels of these key genes were significantly reduced, which aligned with the decreasing trend in alkaloid content. However, a few genes (*5’-NT* and *AMPD* in *LTDC115149*) were significantly upregulated during leaf development, suggesting that they may play important roles in purine recycling, phosphorus reuse, energy homeostasis, and secondary metabolism within the leaves.

### 3.6 Correlation characteristics of major metabolites in tea leaves at different developmental stages

**Figure 7:**
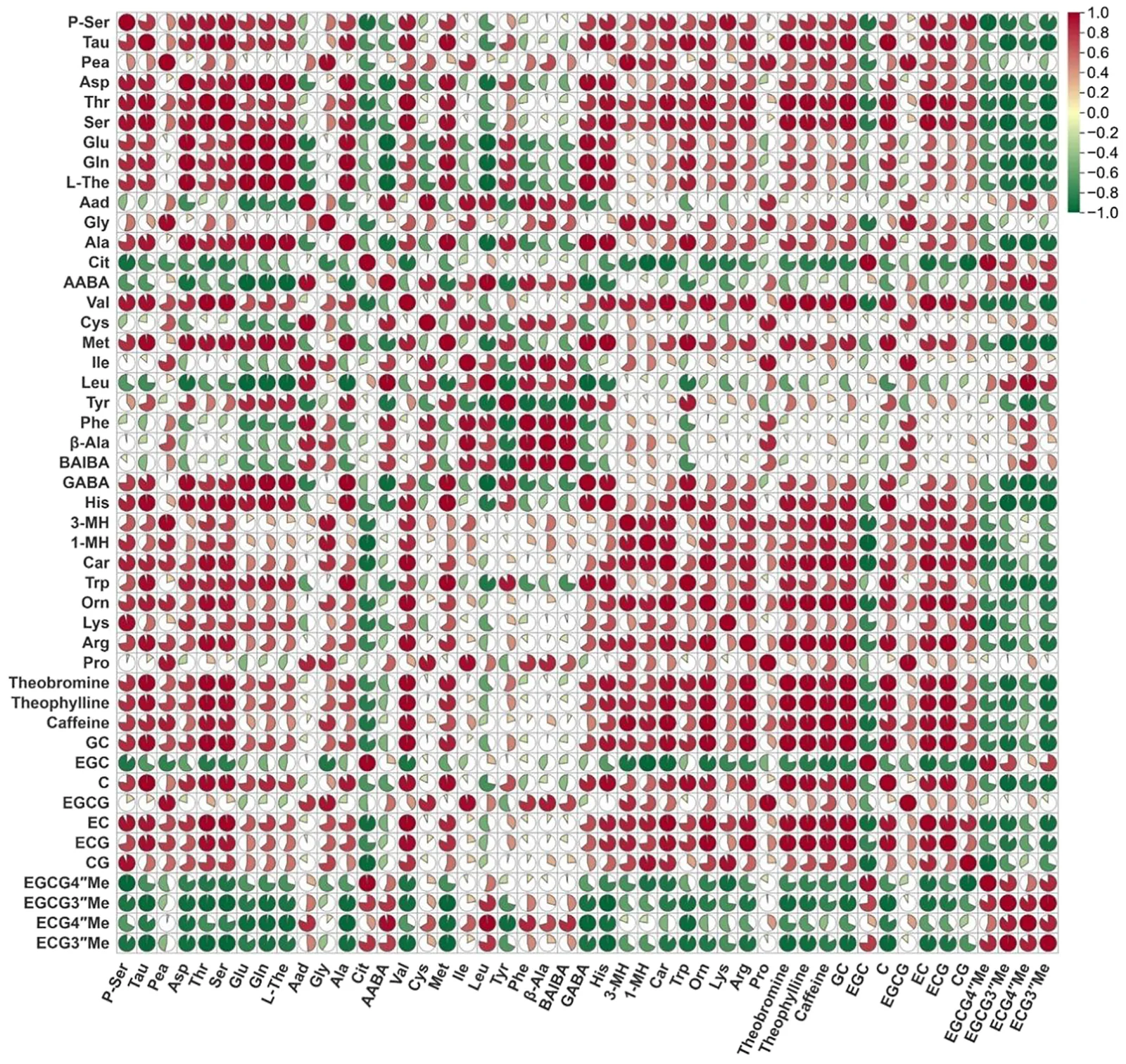
Pearson correlation analysis of the three major quality components (amino acids, alkaloids, and catechins) in tea leaves at different developmental stages. The rows and columns correspond to individual amino acids, alkaloids (caffeine, theobromine, and theophylline), and catechin monomers. The color bar on the right denotes Pearson’s correlation coefficients: red indicates a positive correlation, green indicates a negative correlation, and increased color saturation represents a stronger correlation.

Through correlation analysis, this study systematically elucidated the association patterns among the core quality components (amino acids, alkaloids, and catechins) in buds and leaves at different maturity levels. The correlation heatmap results indicate the potential presence of extensive and significant synergistic or antagonistic relationships among various compounds, providing key insights into the metabolic network regulation underlying the formation of tea quality (**Figure 7**).

Among the interactions between amino acids and other components, amino acids closely associated with the freshness and crispness of tea, such as L-The, Glu, and Gln, were strongly linked to most free amino acids (such as Asp, Ala, and GABA), exhibiting a strong positive correlation (**Figure 7**). This suggests that the pathways for synthesizing L-The, Glu, and Gln in tea plants may be synergistically regulated, jointly driving the high accumulation of amino acids in bud tissues. Concurrently, these amino acids also showed significant positive correlations with alkaloids, such as caffeine, theobromine, and theophylline, indicating that amino acid and alkaloid metabolism may follow a synergistically enhanced regulatory pattern in buds and young leaves.

Within the catechin group, significant positive correlations were generally observed among most catechin components (C, GC, CG, EC, and ECG), indicating that the upstream metabolic flux in the catechin biosynthesis pathway commonly promotes the biosynthesis of different catechin types (**Figure 7**). In contrast, *O*-methylated catechins (such as EGCG3”Me and ECG3”Me) showed weaker correlations with most conventional catechin components and even exhibited negative correlation trends in some comparisons, suggesting that the biosynthesis of methylated catechins follows a relatively independent regulatory pathway, which may be related to the activity of specific methyltransferase enzymes in the leaf tissue.

Most free amino acids (L-The, Glu, Gln, Asp, Ala, and GABA) exhibited a significant positive correlation with catechin components (C, GC, CG, EC, and ECG), indicating that in buds and young leaves, the biosynthesis of catechins and amino acids is not competitive but rather shows synergistic accumulation (**Figure 7**). This synergy jointly shapes the quality foundation of young fresh tea leaves, characterized by “high freshness and a high polyphenol-to-amino acid ratio.” As leaf maturity increases, the correlations among certain components change, reflecting the dynamic remodeling of the metabolic network during leaf development.

### 3.7 Expression analysis of key DEGs during leaf development stages

**Figure 8:**
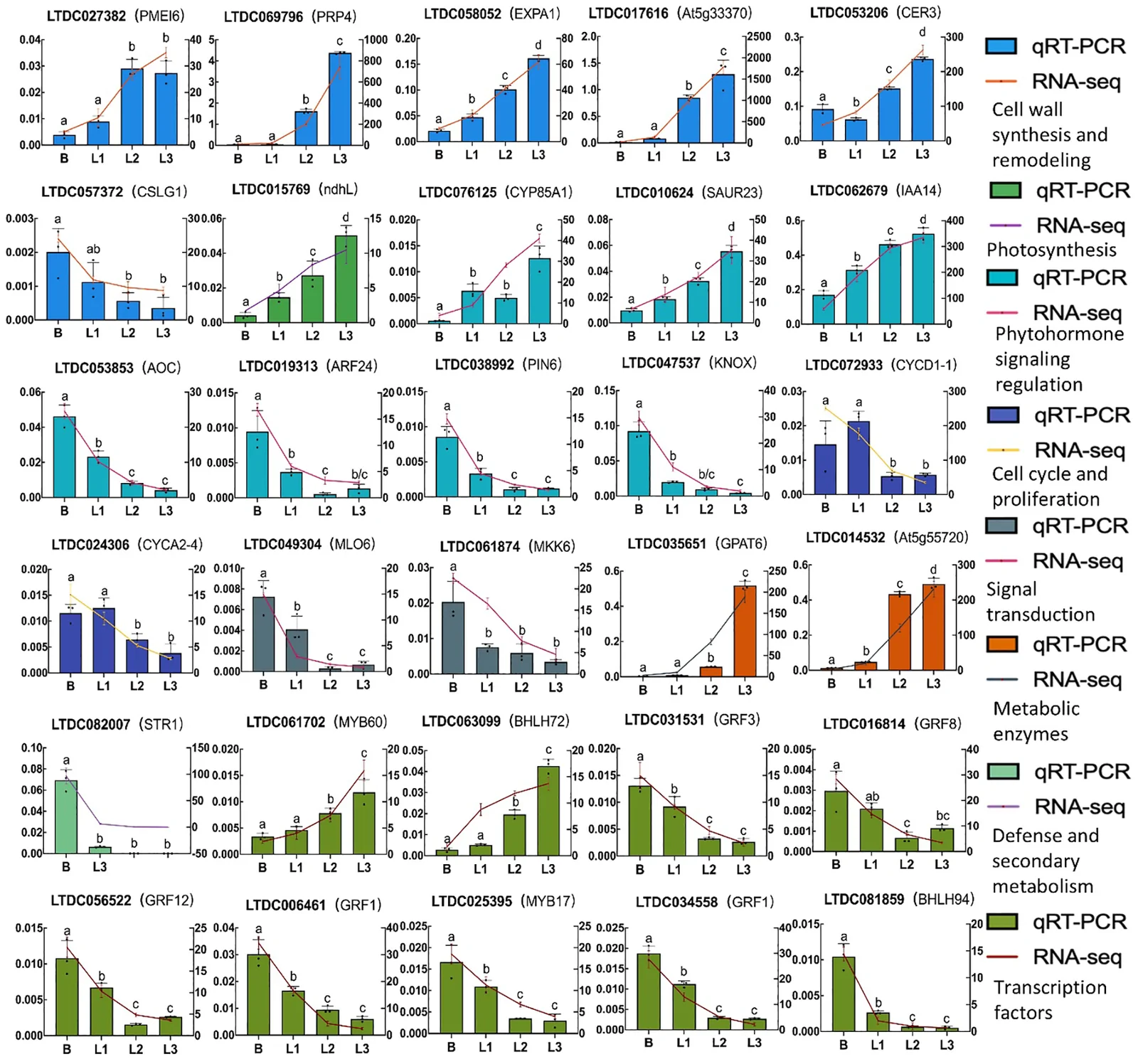
Validation of RNA-seq results was performed using quantitative real-time reverse transcription PCR (qRT-PCR) for key functional genes and transcription factors in tea leaves. Data are presented as mean ± SD (*n* = 3). Means distinguished with different letters are significantly different from each other (*p* ≤ 0.05). B, tea buds; L1, 1st leaf; L2, 2nd leaf; L3, 3rd leaf.

To further identify the key functional genes and transcription factors involved in leaf morphogenesis, 108 leaf development-related DEGs were screened from the transcriptome datasets. Based on functional annotation, the DEGs were mainly involved in pathways including cell wall synthesis and remodeling, photosynthesis, plant hormone signaling regulation, cell cycle, signal transduction, defense and secondary metabolism, and transcription factors (**Figure 8**; **Supplementary Table 2**). qRT-PCR expression validation was performed on 30 highly differentially expressed candidate genes, showing that genes associated with cell wall synthesis (e.g., *EXPA1*, At5g33370, *PRP4*, *PMEI6*), photosynthesis (*ndhL*), and the auxin signaling pathway (e.g., *SAUR23*, *IAA14*) exhibited increasing expression with leaf development. This pattern indicates that during leaf development and maturation, processes such as cell wall formation, photosynthetic system refinement, and auxin-mediated developmental regulation are strongly activated. Conversely, genes related to the cell cycle and proliferation (e.g., *CYCD1–1*, and *CYCA2–4*) showed high expression in B and L1 but were markedly downregulated in L2 and L3, reflecting intense cell division activity in buds and reduced proliferation after leaf maturation. Additionally, genes involved in cell wall synthesis and remodeling (*CsLG1*), hormone signaling (e.g., *KNOX*, *PIN6*, *AOC*, and *ARF24*), signal transduction (e.g., *MKK6* and *MLO6*), and defense (*STR1*) were predominantly expressed in bud tissues. This expression profile suggests that tea plant buds are in a highly active state, characterized by vigorous cell division, morphogenesis, hormonal regulation, signal perception, and stress defense. As the primary organ for new shoot growth, buds exhibit dual molecular traits of rapid development and stress resistance.

Transcription factor genes from families such as *MYB*, *GRF*, and *bHLH* exhibit clear developmental specificity; most are highly expressed in buds (e.g., *GRF1*, *MYB17*, and *bHLH94*), with expression levels declining as leaves mature (**Figure 8**). This pattern suggests that molecular regulation related to secondary metabolism and stress response is more active in buds, consistent with previous findings that quality compounds, such as catechins and amino acids, preferentially accumulate in these tissues. Conversely, transcription factor genes such as *bHLH72* and *MYB60* were significantly upregulated during leaf development, indicating their potential key roles in leaf morphogenesis and functional maturation. Collectively, the qRT-PCR validation reinforced the reliability of the transcriptomic data and revealed the temporal expression dynamics of genes involved in cell proliferation, hormone signaling, cell wall biosynthesis, and secondary metabolism regulation throughout *Camellia sinensis* bud and leaf development. These results provide robust candidate genes and a theoretical foundation for elucidating the molecular regulatory mechanisms underlying tea leaf development and quality.

### 3.8 Cis-acting element analysis of differentially functional genes at various leaf developmental stages

**Figure 9:**
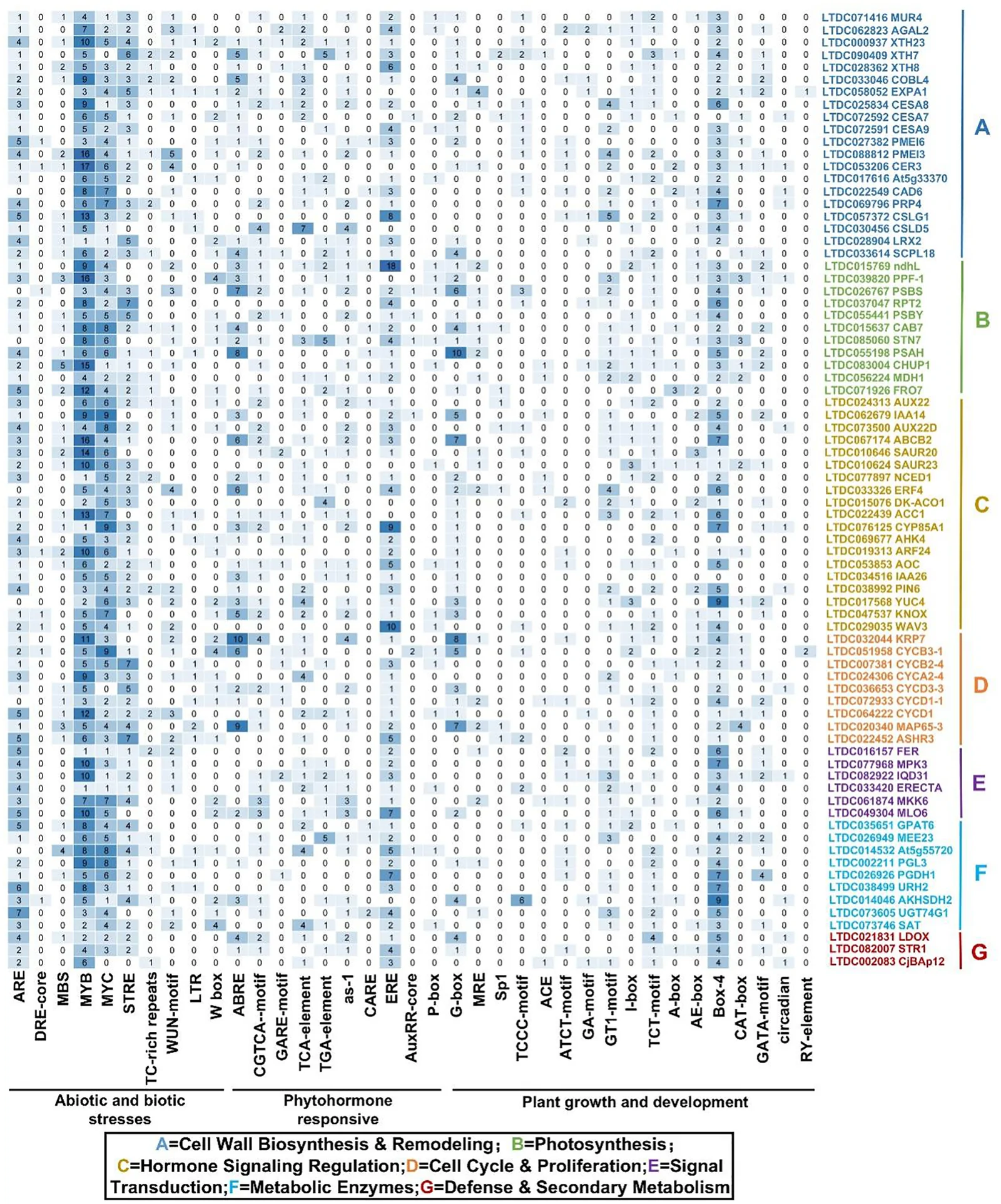
Heatmap showing the enrichment of cis-acting regulatory elements in the promoters of DEGs during tea leaf development. Rows indicate candidate genes, and columns represent the three categories of cis-elements: stress-responsive, hormone-responsive, and growth/development-related elements. Blue denotes the existence of cis-elements, and the color depth corresponds to the element quantity. Categories **(A–G)** represent gene functions: **(A)** cell wall synthesis and remodeling, **(B)** photosynthesis, **(C)** hormone signaling, **(D)** cell cycle and proliferation, **(E)** signal transduction, **(F)** metabolic enzymes, and **(G)** defense and secondary metabolism.

The morphogenesis of *Camellia sinensis* leaves is a complex process synergistically regulated by environmental signals and endogenous hormones, as reflected in the enrichment patterns of cis-acting elements within the promoters of 77 differentially expressed functional genes (B vs. L3). These core genes, involved in cell wall synthesis and remodeling, photosynthesis, hormone signaling, cell cycle, signal transduction, metabolic activity, and defense/secondary metabolism, exhibit promoter regions that are highly enriched with cis-regulatory elements linked to stress responses, hormone signaling, and plant growth, with notable specificity across gene groups (**Figure 9**). Key transcription factor binding sites, such as MYB and MYC elements, with respective counts of 526 and 314. Which are central to abiotic stress responses and morphogenetic processes (Li et al., 2024c), such as epidermal cell differentiation, stomatal closure, and leaf polarity, suggesting a dominant regulatory role for MYB/MYC family factors in tea leaf development. The abundant light-responsive Box4 element (262 pieces) implicates light signaling in chloroplast development, cell elongation, and leaf expansion via photosynthesis gene regulation. Hormone and stress-related elements, including ERE (193 pieces), ARE (182 pieces), and ABRE (125 pieces), indicate the involvement of ethylene, ABA, and oxidative stress pathways in modulating cell division, expansion, and cell wall remodeling, enabling adaptive morphological responses. Gene cluster-specific enrichment patterns further revealed that cell wall-related genes (A) were enriched with TCA, GT1-motif, and WUN-motif elements; photosynthesis genes (B) with GATA-motif and TGA-element; hormone signaling (C) and defense/secondary metabolism (G) genes with TCT-motif and TCA elements; cell cycle genes (D) with W-box; and signal transduction genes (E) with As-1 elements. These findings collectively support a model in which bud and leaf development were orchestrated by the integrated action of hormonal, light, and stress signaling pathways. These cis-regulatory elements mediate the precise temporal and spatial expression of downstream genes, providing critical evidence for the transcriptional regulatory networks governing tea leaf morphogenesis and the biosynthesis of flavor compounds.

## 4 Discussion

**Figure 10:**
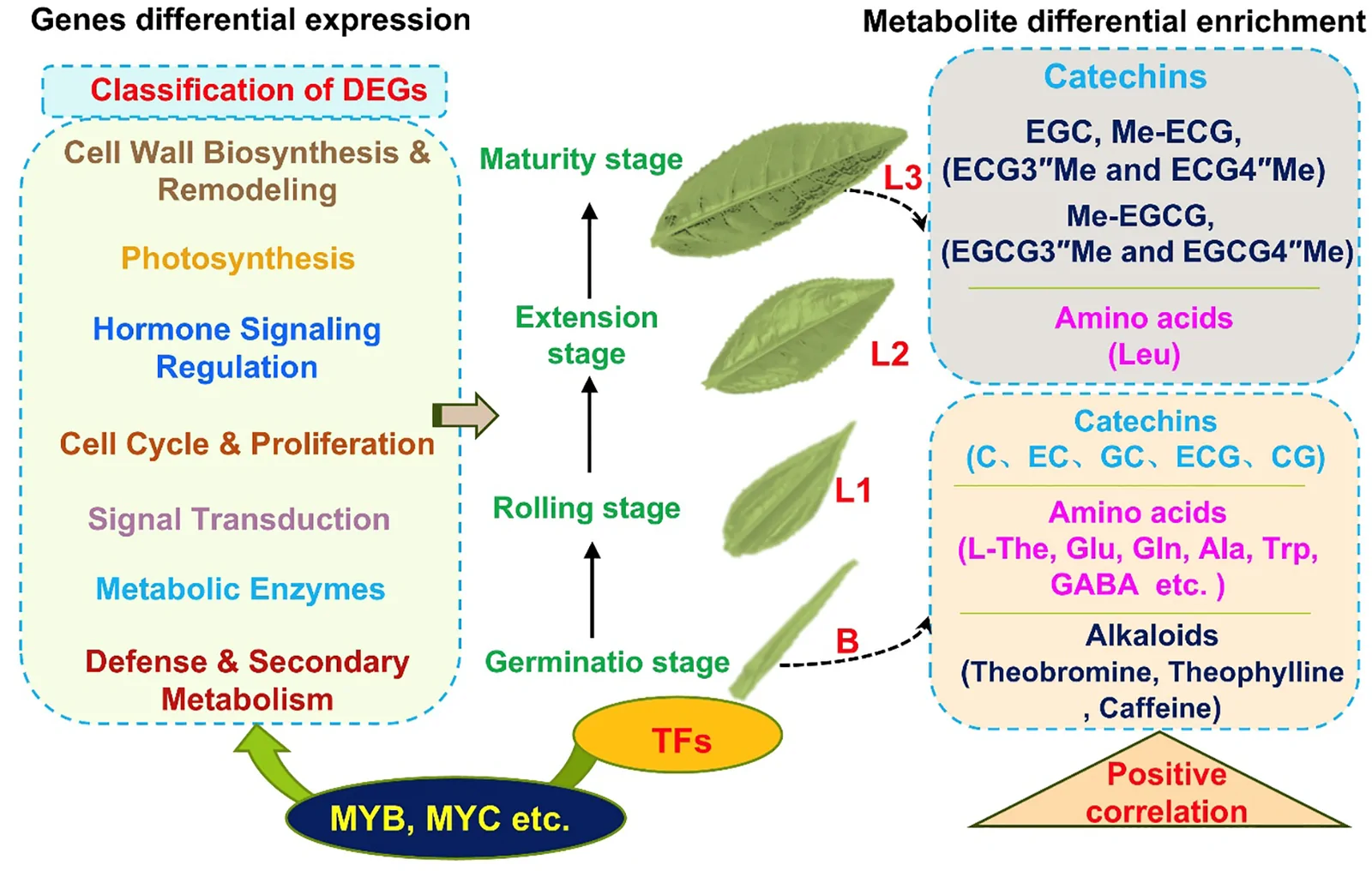
A proposed working model illustrating the dynamic regulatory network of leaf morphogenesis in *Camellia sinensis* revealed by transcriptomic and biochemical analyses.

In this study, we used buds, first leaves, second leaves, and third leaves from tea plants to systematically analyze the dynamic regulatory network governing leaf morphogenesis through transcriptomic and biochemical metabolic analyses. We elucidated the temporal accumulation patterns of key quality metabolites (catechins, amino acids, and alkaloids) and their underlying genetic regulatory mechanisms, thereby deepening our understanding of the synergistic relationship between tea leaf development and quality (**Figure 10**).

Plant leaf development is a biological process characterized by tightly coupled sequential morphogenesis and functional stabilization of leaves. This process not only reflects the plant’s growth status but also represents a critical biological feature of leaf functional maturation, including photosynthesis, metabolic balance maintenance, and environmental response and adaptation (Challa et al., 2021; Li et al., 2024a; Wu et al., 2025a). In this study, PCA and differential gene expression analyses revealed that transcriptomic differences between buds and leaves progressively increased throughout development, with significant enrichment of metabolic pathway-related genes. These findings indicate that the morphological maturation of tea leaves is accompanied by extensive transcriptional reprogramming, predominantly involving coordinated changes in both secondary and primary metabolism. This pattern aligns with previously reported phase differentiation observed in transcriptomic and metabolomic studies of tea plant buds and leaves (Chen et al., 2025; Xu et al., 2026; Zhao et al., 2026) and is consistent with the general developmental regulatory mechanisms characterized by dynamic transcriptome remodeling and pathway-specific metabolic activation during leaf development in model plants, such as rice (Wu et al., 2025b) and *Arabidopsis* (Omidbakhshfard et al., 2021). By encompassing the entire developmental trajectory from bud to leaf, this study elucidated the regulatory continuity governing the full cycle of tea leaf development.

This study revealed that the total catechin content follows a developmental pattern characterized by high levels in tender buds and young leaves, decreasing as leaves mature, with EGCG identified as the predominant catechin. Methylated catechins show relative enrichment in mature leaves, corroborating the regulatory role of leaf maturity in catechin biosynthesis and accumulation (Yao et al., 2025; Wu et al., 2025a; Luo et al., 2025). Interestingly, most catechin monomers (C, EC, GC, ECG, CG, and EGCG) in L3 were significantly less abundant than in B, whereas the accumulation of *O*-methylated catechins (Me-ECG and Me-EGCG) was greatly enhanced, which balanced the total catechin levels of B and L3. Transcriptomic analysis has demonstrated that key genes in the phenylpropanoid and flavonoid biosynthetic pathways, including *CHS*, *LAR*, and *ANS*, exhibit elevated expression in tender shoots, underscoring their critical functions in catechin production (Jiang et al., 2025). Additionally, genes such as *DFR*, *LAR*, and *ANR* are upregulated in mature leaves, indicating enhanced metabolic activity related to flavonoid and catechin biosynthesis at this stage (Ying et al., 2017). Notably, the genes responsible for methylated catechin biosynthesis, *CsFAOMT1*, *CsFAOMT2*, and *CCoAOMT*, were significantly upregulated in mature leaves, consistent with their specific catalytic roles in this pathway (Jin et al., 2023). These results suggest that tea plants orchestrate the developmentally regulated temporal expression of structural genes to achieve stage-specific catechin composition. This regulation sustains a high phenolic content in young leaves, balancing economic value and stress resistance, while promoting distinctive methylation-related metabolic traits in mature leaves, providing a molecular basis for the unique accumulation of endogenous compounds in tea plants.

Amino acid metabolism analysis revealed that umami-related amino acids, including L-The, Glu, and Gln, were significantly enriched in buds and declined as leaves matured. Key genes associated with ammonium assimilation (*GS* and *GOGAT*) and theanine synthetase (*TS*) exhibit high expression levels in buds, supplying abundant precursors and enzymatic capacity for amino acid biosynthesis. These results indicate that buds are the principal sites for amino acid biosynthesis and storage in tea plants. This pattern aligns with the general tendency for preferential amino acid accumulation in young tissues (Zhang et al., 2025b; Zhao et al., 2026) and clarifies, at the transcriptomic level, the stage-specific activation of theanine biosynthetic pathways, presenting crucial targets for enhancing tea freshness. Furthermore, free amino acids such as urea, Asn, Amm, and Hyp were undetected in buds and leaves, which may reflect their specific synthesis, transport, and degradation dynamics (Yu et al., 2024).

Purine alkaloids, including caffeine, theobromine, and theophylline, predominantly accumulate in buds and young leaves, with levels sharply declining as leaves mature. The expression profiles of key caffeine biosynthesis genes, *XMT*, *MXMT*, and *TCS*, along with upstream purine metabolism genes such as *ADK* and *IMPDH*, closely mirror these metabolite dynamics, indicating tight transcriptional regulation consistent with the metabolite accumulation patterns (Zhang et al., 2022). Notably, theacrine, a purine alkaloid enriched in specialized tea varieties such as Kucha, was absent in the LTDC cultivar, corroborating our metabolomic findings (Wu et al., 2026). Functionally, elevated caffeine content in tender leaves contributes to enhanced insect resistance and stress tolerance, while underpinning the characteristic flavor profile of tea. This study clarifies the molecular mechanisms underlying the developmental regulation of purine alkaloid biosynthesis, consistent with previous research on alkaloid dynamics in tea plants (Jia et al., 2026).

Correlation analysis revealed that amino acids, alkaloids, and certain catechins were synergistically and positively correlated in young tissues, indicating that buds and young leaves exhibit active anabolic metabolism with concurrent enhancement of multiple metabolic pathways. This finding further supports the existence of a dynamic regulatory network among metabolites in tea plants, encompassing processes such as amino acid synthesis and transport, along with flavonoid biosynthesis (Feng et al., 2026; Zhang et al., 2026). Conversely, methylated catechins displayed weak correlations with conventional catechins, suggesting the operation of relatively independent biosynthetic and regulatory pathways (Jin et al., 2023). This “predominantly synergistic with subsidiary specificity” metabolic network represents a crucial adaptive strategy for tea plants to balance growth, defense, and quality formation, providing a metabolic basis for the superior quality of young raw tea leaves.

Cis-element analysis of key functional genes involved in leaf morphogenesis revealed that tea leaf development is co-regulated by hormone signals, including auxin, abscisic acid, and gibberellin, alongside light and stress signals (McAllister et al., 2025). Transcription factor families, such as MYB, bHLH, WRKY, and TCP, form interconnected regulatory networks that integrate leaf morphogenesis with secondary metabolism (Li et al., 2022; Yu et al., 2021; Liu et al., 2024; Peng et al., 2025). The promoter regions of these genes are enriched with cis-elements, such as ABRE, AuxRR-core, and G-box, which mediate precise transcriptional responses to hormonal and environmental cues. These findings refine the hierarchical regulatory framework of tea leaf development, characterized by an “environment–hormone–transcription factor–structural gene–metabolite” cascade, consistent with established mechanisms, such as the MBW complex and hormone–transcription factor networks (Xu et al., 2026).

This study has certain limitations, primarily focusing on natural developmental gradients. First, all plant materials used in this study were obtained from a single tea cultivar (LTDC). Considering the extensive genetic variation among tea germplasms, the developmental metabolic characteristics and transcriptional regulatory networks uncovered in the present study may not be universally generalizable to other tea accessions with divergent metabolic profiles and sensory qualities. Second, leaf samples ranging from buds to the 3^st^ leaf were harvested during a single growing season. Fluctuations in seasonal factors, including temperature, light intensity, precipitation, and soil nutrient supply, profoundly influence the biosynthesis of secondary metabolites and genome-wide transcriptional reprogramming in tea leaves (Wu et al., 2025a). Therefore, multi-season sampling across consecutive years is necessary to eliminate seasonal confounding effects and further verify the developmental accumulation patterns determined in this study. Third, multi-omics integration of the transcriptome and metabolome was performed based on correlation analysis only. Although multiple prominent gene–metabolite correlation pairs associated with key flavor components were identified, statistical correlation alone is insufficient to establish direct causal regulatory interactions. Coordinated changes in gene expression and metabolite abundance may arise from common upstream developmental cues rather than one-to-one direct regulatory effects of structural genes on metabolite synthesis (Ni et al., 2025; Fàbregas et al., 2026). In addition to the limitations of the experimental design, this investigation merely characterizes intrinsic ontogenetic variation along leaf developmental gradients without incorporating exogenous environmental stimuli or agronomic management treatments. Therefore, future research should incorporate multi-cultivar comparative analysis and continuous multi-season sampling to test the universality of the developmental metabolic regulatory network, as well as incorporate environmental and cultivation factor treatments, along with transgenic functional validation of candidate key genes (McAllister et al., 2025). Techniques such as gene editing and transient expression can be employed to clarify the functions of these core regulatory genes (Nagalakshmi et al., 2026; Mohajer et al., 2023). Moreover, integrating environmental factors, including light, temperature, fertilization, and pruning, will enable the exploration of their regulatory effects on the developmental-metabolic network, thereby establishing a more comprehensive theoretical foundation for precision cultivation and quality-oriented management in tea plantations. This study systematically elucidates the transcriptional-metabolic synergistic regulatory mechanisms underlying leaf morphogenesis in tea plants, clarifies developmental allocation patterns and core genes associated with key quality-related compounds, enriches the theoretical framework of tea plant developmental biology and quality formation, and holds significant practical value for high-quality, efficient cultivation, variety improvement, and optimization of harvest timing for raw materials.

## Data Availability

The original contributions presented in the study are included in the article/**Supplementary Material**. Further inquiries can be directed to the corresponding authors.
